# Utilization and determinants of rehabilitation in patients receiving extracorporeal membrane oxygenation in South Korea (2014–2018): A study based on Health Insurance Review and Assessment (HIRA) data

**DOI:** 10.1097/MD.0000000000049196

**Published:** 2026-06-05

**Authors:** Hyun-Seok Jo, Min-Keun Song, Jae-Young Han, In-Sung Choi, Hyeng-Kyu Park

**Affiliations:** a Department of Physical and Rehabilitation Medicine, Regional Cardiocerebrovascular Center, Center for Aging and Geriatrics, Chonnam National University Medical School and Hospital, Gwangju, Republic of Korea; b Department of Physical and Rehabilitation Medicine, Chonnam National University Hospital, Gwangju, Republic of Korea.

**Keywords:** extracorporeal membrane oxygenation, intensive care, rehabilitation

## Abstract

Although the use of extracorporeal membrane oxygenation (ECMO) is increasing globally, survivors often face long-term physical and functional impairments. While recent clinical shifts toward “awake ECMO” highlight the importance of early rehabilitation, there is a significant gap between clinical evidence and real-world practice. This study used nationwide data to investigate the rates of rehabilitation utilization among ECMO patients in South Korea and identify the clinical and regional factors influencing access to rehabilitation. This retrospective cohort study focused on rehabilitation interventions from 2014 to 2018. Patients aged between 20 and 90 years who had received ECMO treatment, based on the Korean Health Insurance Review and Assessment database, were selected. Cases with hospital stays of <3 days were excluded. Patients admitted for respiratory illness were more likely to undergo rehabilitation than those admitted for circulatory disease (adjusted odds ratio [aOR]: 1.656, 95% confidence interval [CI]: 1.242–2.207). Patients who received ECMO for >4 days were more likely to receive rehabilitation (aOR for 5–9 days: 2.740, 95% CI: 2.094–3.587; aOR for ≥10 days: 2.987, 95% CI: 2.179–4.094). Patients on mechanical ventilation for >4 days were less likely to receive rehabilitation (aOR: 0.003, 95% CI: 0.002–0.005). Those with an intensive care unit length of stay exceeding 7 days were more likely to undergo rehabilitation (aOR: 7.787, 95% CI: 6.010–10.090). Regions with more hospitals offering cardiac rehabilitation programs, including cardiocerebrovascular centers, were positively associated with rehabilitation uptake. Rehabilitation in ECMO patients is influenced by clinical severity as well as structural and regional factors. Specifically, regional disparities in infrastructure and varying physician perceptions across specialties create inequalities in care access. Our findings underscore the need for standardized rehabilitation protocols and targeted policy interventions to harmonize clinical practices and optimize long-term functional recovery among ECMO survivors nationwide.

## 1. Introduction

Extracorporeal membrane oxygenation (ECMO) is a critical therapeutic intervention for intensive care unit (ICU) patients with cardiac and/or respiratory failure.^[1]^ The use of ECMO has been rising globally,^[2-5]^ with a further surge during the COVID-19 pandemic.^[6,7]^ Patients who survive ECMO-related illnesses often experience a diminished health-related quality of life, including in physical, mental, functional, and social well-being.^[8]^ These challenges correlate with elevated healthcare costs and extended periods of unemployment.^[9]^ Therefore, addressing the functional recovery of ECMO survivors is a pressing public health priority.

A structured rehabilitation is essential for mitigating these challenges. Early rehabilitation reduces the incidence of ICU-acquired weakness,^[10]^ and early mobility is associated with lower readmission and mortality rates.^[11]^ Post-ICU rehabilitation also plays a significant role in enhancing recovery. Park et al reported that rehabilitation of ICU survivors during hospitalization considerably reduced the risk of 30-day ICU readmission and emergency room visits.^[12]^ Several factors, including sedation, consciousness level, ECMO configuration, cannula type, and cannulation site, influence the feasibility of rehabilitation in patients on ECMO.^[13]^ Research on patients receiving ECMO remains limited despite the importance of rehabilitation. Historically, patients on venovenous or venoarterial ECMO were deemed unsuitable for mobilization therapy because of the use of sedatives or neuromuscular blocking agents. However, recent clinical paradigm shifts, driven by advancements in cannulation techniques and “awake ECMO” protocols, have demonstrated that mobilization is feasible and beneficial for these high-acuity patients.^[14-16]^ Despite these clinical advancements, a significant gap remains between evidence-based recommendations and real-world practice, particularly at the national level. Most previous studies are limited to single-center experiences or small case series, which fail to capture the broader rehabilitation delivery landscape. While the technical application of ECMO has matured in South Korea, the current status of subsequent rehabilitation, including its utilization rate and the sociodemographic factors that influence access, is poorly understood. Recent international survey data have confirmed that early physiotherapy is feasible for ECMO patients. However, significant challenges remain, including the absence of standardized protocols and inadequate staffing.^[17]^ Furthermore, regional medical resource disparities may create rehabilitation uptake inequalities, a factor that has not been sufficiently explored in previous studies. To address these gaps, this study used the Health Insurance Review and Assessment (HIRA) nationwide database to comprehensively analyze rehabilitation in patients on ECMO in South Korea. The objective was to investigate the clinical and demographic factors that determine rehabilitation outcomes and to elucidate the status and trends of ECMO-related rehabilitation. This study sought to provide the empirical evidence necessary to optimize recovery pathways and inform healthcare policy for this vulnerable population by identifying the determinants of rehabilitation utilization.

## 2. Materials and methods

### 2.1. Data source

This study used South Korea’s HIRA database. Data were collected from January 1, 2014 to December 31, 2018. In South Korea, all diagnoses and prescriptions are registered in the HIRA database, and the entire population is covered by the National Health Insurance Service or the Medical Aid Program.^[18]^ The HIRA database undergoes a review process for claims submitted for reimbursement to the Korean National Health Insurance. Therefore, the database includes virtually all medical admissions, except for those deemed inappropriate by HIRA and rejected for payment. Notably, the Korean government’s insurance programs cover approximately 97% of the population, while the Medical Aid Program covers the remaining 3%, who cannot afford national insurance.^[19]^ Hence, the database includes data on nearly all inpatient and outpatient cases in hospitals across South Korea.

### 2.2. Study population

**Figure 1.**
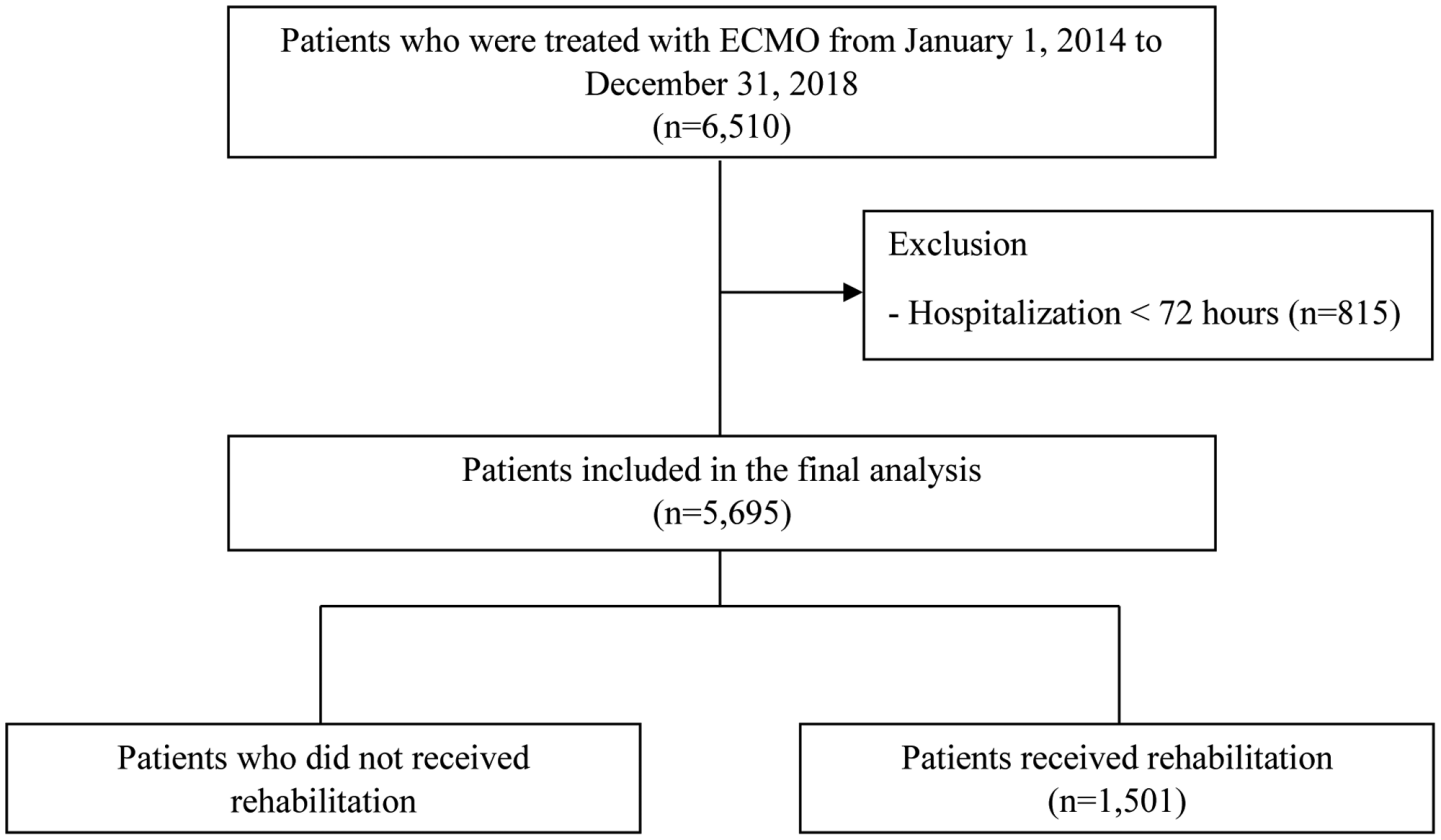
Flow chart illustrating the selection process for patients receiving ECMO. ECMO = extracorporeal membrane oxygenation.

This study’s dataset included patients aged between 20 and 90 years, who had been treated with ECMO (N = 6510). Patients with hospital stays <3 days were excluded (N = 815). The study population was divided into 2 groups based on the presence or absence of rehabilitation during hospitalization. Of the patients treated with ECMO, those who received rehabilitation were categorized into the rehabilitation group, while the remaining patients were classified as the non-rehabilitation group. The study included 5695 patients who met the eligibility criteria (Fig. 1).

### 2.3. Measurements

This study’s variables were selected to include both clinical determinants and regional factors, providing a comprehensive view of rehabilitation utilization. Regional factors were included to examine the structural characteristics and geographical distribution of healthcare delivery in South Korea.

ECMO support was defined using mandatory claim codes (Table S1, Supplemental Digital Content) from the HIRA database. The presence of rehabilitation was also determined through claim codes required for hospital cost submissions to HIRA during inpatient stays. Rehabilitation therapies included physical (MM101, MM102, MM105, MM301, MM302) and occupational (MM111, MM112, MM113, MM114) therapies.

Data on demographic characteristics, prescriptions, procedures, and diagnoses were obtained through HIRA claim codes (Tables S1 and S2, Supplemental Digital Content). Diagnoses were recorded in accordance with the Korean Classification of Diseases (6th edition), which is a modified version of the International Classification of Diseases (10th edition).^[20]^

Procedures identified through HIRA claim codes included mechanical ventilation (MV), hemodialysis, continuous renal replacement therapy, and ECMO. MV and ECMO usage were categorized based on duration. The utilization of vasopressor drugs, neuromuscular blockers, and sedatives was identified through Korean Drug and Anatomical Therapeutic Chemical Codes (Table S2, Supplemental Digital Content).^[21]^ Hospital regions were categorized by grouping geographically proximate locations.

### 2.4. Statistical analyses

Patients were stratified into 2 groups based on the presence or absence of rehabilitation during their hospital stay. Variable distribution was described using means and standard deviations. Chi-square tests were used for categorical variables, and Student *t* tests were used for continuous variables.

Logistic regression analysis was used to identify univariate and multivariable predictors of rehabilitation. Odds ratios (OR) were calculated with 95% confidence intervals (CI). A *P*-value of <.05 indicates statistically significant differences. Statistical analyses were performed using SAS software.

### 2.5. Ethics statement

The study was conducted in accordance with the ethical standards of the Declaration of Helsinki. All patient identification information was removed by HIRA before the data were provided. Ethical approval for the study protocol was granted by the Institutional Review Board of Chonnam National University Hospital (IRB No. CNUH-EXP-2022-215), which waived the requirement of informed consent because of the study’s retrospective design and anonymity of the HIRA database. Official approval to access and utilize data from December 26, 2022 to May 24, 2023, was obtained from the HIRA Research Inquiry Commission (No. M20220722003).

## 3. Results

A total of 5695 patients met our inclusion criteria in the period between January 1, 2014 and December 31, 2018. The clinicopathological and demographic characteristics of the patients who received ECMO are presented in Tables 1 and 2. An unadjusted comparison revealed a statistically significant primary diagnosis difference between the group that received rehabilitation and the 1 that did not (*P* < .01). Circulatory disease was the most prevalent diagnosis in both groups (47.4% vs 64.5%), but the rehabilitation group had a higher proportion of patients with respiratory disease (25.2% vs 14.9%, Table 1). The median ECMO duration was notably longer among those who received rehabilitation (10 vs 6 days, *P* < .01), while the median duration of MV was shorter (2 vs 9 days, *P* < .01; Table 2). Additionally, the median ICU length of stay (LOS; 24 vs 11 days, *P* < .01) and the median hospital length of stay (LOS; 32 vs 15 days, *P* < .01) were longer in the rehabilitation group. There was no statistically significant difference in the use of sedatives, vasopressors, or neuromuscular blockers between the groups, except for hemodialysis (Table 2).

**Table 1:** Characteristics of patients with ECMO treatment according to rehabilitation usage in South Korea, January 2014 to December 2018.

Variables	Total (N = 5695)	Rehabilitation (N = 1501)	Non-rehabilitation (N = 4194)	*P*-value
Age (yr), median (IQR)	54 (37–70)	52 (38–66)	54 (37–71)	<.001
Sex				.033
Male	3773 (66.3)	961 (64.0)	2812 (66.3)	
Female	1922 (33.7)	542 (36.0)	1382 (33.0)	
Primary diagnosis				<.001
Cardiovascular disease	3377 (59.3)	712 (47.4)	2665 (63.6)	
Respiratory disease	1001 (17.6)	378 (25.2)	623 (14.9)	
Neoplasm	417 (7.3)	127 (8.5)	290 (6.9)	
Gastrointestinal disease	113 (1.8)	36 (2.4)	77 (1.8)	
Infectious disease	105 (1.8)	32 (2.1)	73 (1.7)	
Others	678 (11.9)	216 (14.4)	462 (11.0)	
Region				<.001
Seoul	2794 (49.1)	867 (57.8)	1927 (45.9)	
Gyeonggi	843 (14.8)	185 (12.3)	658 (15.7)	
Incheon	321 (5.6)	44 (2.9)	277 (6.6)	
Gangwon	72 (1.3)	14 (0.9)	58 (1.4)	
Daegu-Gyeonbuk	327 (5.7)	60 (4.0)	267 (6.4)	
Gwangju-Jeonnam	339 (6.0)	60 (4.0)	279 (6.7)	
Jeonbuk	102 (1.8)	25 (1.7)	77 (1.8)	
Daejeon-Chungnam Chungbuk	223 (3.9)	25 (1.7)	198 (4.7)	
Bu-Ul-Gyeong	674 (11.8)	221 (14.7)	453 (10.8)	

All values except for age are presented as frequency (%).

Bu = Busan, Gyeong = Gyeongnam, ECMO = extracorporeal membrane oxygenation, IQR = interquartile range, Ul = Ulsan.

**Table 2:** Procedures and hospital outcomes of ECMO patients according to rehabilitation in South Korea, January 2014 to December 2018.

Procedures & hospital outcomes	Total (N = 5695)	Rehabilitation (N = 1501)	Non-rehabilitation (N = 4194)	*P*-value
Mechanical ventilation				<.001
1–4 d	3059 (53.7)	1427 (95.1)	1632 (38.9)	
> 4 d	2636 (46.3)	74 (4.9)	2562 (61.1)	
ECMO				<.001
≤4 d	2634 (46.3)	510 (34.0)	2124 (50.6)	
5–9 d	1645 (28.9)	428 (28.5)	1217 (29.0)	
≥10 d	1416 (24.9)	563 (37.5)	853 (20.3)	
Sedatives (d), median (IQR)	10 (0–22)	11 (0–22)	10 (0–21)	.244
Vasopressors (d), median (IQR)	10 (0–22)	10 (0–21)	10 (0–22)	.545
Neuromuscular blockers (d), median (IQR)	6 (0–12)	6 (0–13)	6 (0–12)	.860
Hemodialysis (d), median (IQR)	10 (0–20)	7 (0–14)	17 (4–29)	<.001
ICU LOS (d), median (IQR)	14 (1–27)	24 (9–38)	11 (1–21)	<.001
≤7 d	2669 (46.9)	353 (23.5)	2316 (55.2)	
>7 d	3026 (53.1)	1148 (76.5)	1878 (44.8)	
Hospital LOS (d), median (IQR)	20 (4–35)	32 (14–51)	15 (3–27)	<.001

All values except for age are presented as frequency (%) except where indicated.

ECMO = extracorporeal membrane oxygenation, IQR = interquartile range, LOS = length of stay.

Multivariable analysis revealed that patients admitted for respiratory disease had a higher likelihood of receiving rehabilitation when compared with those admitted for circulatory disease (adjusted OR [aOR]: 1.656, 95% CI: 1.242–2.207; Table 3). Patients who received ECMO for >4 days were more likely to receive rehabilitation (aOR for 5–9 days: 2.740, 95% CI: 2.094–3.587; aOR for ≥10 days: 2.987, 95% CI: 2.179–4.094). Those who were on MV for >4 days were less likely to undergo rehabilitation (aOR: 0.003, 95% CI: 0.002–0.005). Patients with an ICU LOS of >7 days had a higher likelihood of receiving rehabilitation when compared with those with a LOS of 7 days or less (aOR: 7.787, 95% CI: 6.010–10.090). In regional comparisons, hospitals in Seoul (aOR: 2.110, 95% CI: 1.285–3.467) and Bu-Ul-Gyeong (aOR: 3.796, 95% CI: 2.186–6.590), which had more hospitals offering cardiac rehabilitation programs, including regional cardiocerebrovascular centers, were more likely to provide rehabilitation.

**Table 3:** Multivariable regression for rehabilitation of patients with ECMO treatment in South Korea.

Variables	Odds ratio (95% CI)	*P*-value
Age	1.038 (0.984–1.096)	.174
Sex		
Male	1 (reference)	
Female	1.038 (0.838–1.287)	.731
Primary diagnosis		
Cardiovascular	1 (reference)	
Respiratory	1.656 (1.242–2.207)	**.041**
Neoplasm	0.770 (0.520–1.140)	.192
Gastrointestinal	0.979 (0.493–1.942)	.952
Infectious	2.273 (0.992–5.207)	.052
Others	1.096 (0.797–1.508)	.572
ECMO duration		
1–4	1 (reference)	
5–9	2.740 (2.094–3.587)	**<.001**
≥10	2.987 (2.179–4.094)	**<.001**
MV duration		
1–4	1 (reference)	
>4	0.003 (0.002–0.005)	**<.001**
ICU LOS		
≤7	1 (reference)	
>7	7.787 (6.010–10.090)	**<.001**
Hospital LOS	1.088 (1.078–1.097)	**<.001**
Region		
Gwangju, Jeonnam	1 (reference)	
Seoul	2.110 (1.285–3.467)	**.003**
Gyeonggi	1.622 (0.929–2.830)	.089
Incheon	0.685 (0.348–1.348)	.273
Gangwon	2.425 (0.851–6.915)	.097
Daegu-Gyeongbuk	1.603 (0.818–3.144)	.169
Jeonbuk	2.040 (0.812–5.125)	.129
Daejeon-Chungnam, Chungbuk	0.994 (0.459–2.153)	.987
Bu-Ul-Gyeong	3.796 (2.186–6.590)	**.017**

Bold indicates the same level of statistical significance.

AUC = area under the curve, Bu = Busan, CI = confidence interval, ECMO = extracorporeal membrane oxygenation, Gyeong = Gyeongnam, ICU = intensive care unit, LOS = length of stay, MV = mechanical ventilation, Ul = Ulsan.

## 4. Discussion

In this analysis of Korean insurance data, the prevalence of rehabilitation of patients on ECMO was associated with clinicopathologic and demographic factors. Although the clinical predictors of rehabilitation in our cohort are consistent with earlier studies, this analysis reveals less-explored significant regional disparities and practical barriers. Patients with longer ICU LOS were more likely to receive rehabilitation services. Extended LOS in the ICU is associated with a higher risk of ICU-acquired weakness,^[9,22]^ necessitating rehabilitation. However, resources for rehabilitation are limited relative to the patient population with critical illnesses in Korea. Moreover, the focus is primarily on acute care in the early phase of ICU admission, resulting in fewer rehabilitation referrals in the initial hospitalization period. The number of rehabilitation referrals tends to increase subsequently. Patients requiring MV are also at an increased risk of physical and psychological complications, which contribute to prolonged weaning from MV and increased morbidity and mortality.^[23]^ Thus, longer weaning periods necessitate extended ICU stays, which lead to an increased demand for rehabilitation.

Contrary to these trends, our data indicate that MV exceeding 4 days was a negative predictor for rehabilitation, with these patients receiving fewer rehabilitation services, even when accounting for equivalent ICU LOS. This suggests that the MV apparatus may be a barrier to rehabilitation. Studies have shown that for mechanically ventilated patients, rehabilitation is safe and beneficial, and it improves physical and psychological functioning, as well as aids the weaning process.^[23,24]^ Our results suggest that awareness is needed for a prevailing belief that rehabilitation may be risky and inadvisable for ventilated patients.

We also observed that patients with a longer hospital LOS were more likely to receive rehabilitation. A previous study reported that rehabilitation did not significantly reduce hospital LOS for critically ill patients.^[25]^ Patients requiring intensive medical care are more likely to have extended tertiary hospital stays, potentially increasing the demand for rehabilitation services. Therefore, metrics like emergency room visits or ICU readmissions post-discharge may offer a more accurate comparison than simply evaluating rehabilitation against hospital LOS. Considering these metrics, studies on the rehabilitation of patients on ECMO are necessary.

A study by Smith et al associated ECMO use for 4 days or less with increased mortality.^[26]^ In our analysis, patients on ECMO for >4 days were more likely to receive rehabilitation. Given the longer duration of ECMO application, the mortality rate appears lower than in patients with a shorter period of ECMO. Consequently, it is anticipated that patients with an extended ECMO duration have more opportunities for rehabilitation therapy. However, research on the impact of ECMO duration on rehabilitation is limited, and studies are needed to comprehensively explore this aspect.

Our results show that patients with respiratory disease received more frequent rehabilitation prescriptions. In the Korean healthcare system, referrals for rehabilitation treatment are issued by physicians across various departments. Given that these referrals are made by physicians, they play a pivotal role in the rehabilitation process of patients supported by ECMO. A study by Abrams et al found that the rate and nature of such referrals are impacted by multiple factors, including the environmental context, knowledge, attitudes, and behaviors.^[27]^ Notably, the understanding of the referral process by clinicians was identified as a significant contributor.^[27]^ Our findings suggest that there is a disparity in physicians’ perceptions about the necessity of rehabilitation for patients on ECMO. This underscores the need for targeted interventions to harmonize the understanding and perspectives of physicians about rehabilitation.

Finally, there were regional differences in rehabilitation prescriptions for patients on ECMO. Specifically, regions with a greater prevalence of hospitals offering cardiac rehabilitation programs, including regional cardiocerebrovascular centers, had higher rehabilitation prescription rates.^[28]^ Another study observed a similar trend, noting that patients transferred from spinal cord injury trauma centers to rehabilitation facilities were less likely to be readmitted compared to those from non-spinal cord injury trauma centers.^[29]^ These findings highlight the importance of healthcare infrastructure in shaping rehabilitation practices and suggest that infrastructure gaps could affect the rehabilitation of ECMO-supported patients.

This study has limitations. First, the use of data from insurance reimbursement claims precluded the precise identification of rehabilitation service timing and the severity of patients’ conditions. This made it difficult to determine whether rehabilitation services were preventive or for functional recovery. Second, our dataset is from a national health insurance system, which may limit the generalizability of our findings to other countries with different insurance frameworks. Nonetheless, there is a paucity of large-scale data on the rehabilitation of patients who undergo ECMO. Our study contributes valuable insights for future research on ECMO rehabilitation by evaluating all such patients. Additionally, we identified several factors influencing rehabilitation outcomes and differences between the rehabilitation and non-rehabilitation groups.

## 5. Conclusions

Our findings suggest that various physical and clinicopathological conditions, as well as regional differences, are associated with rehabilitation outcomes in patients who receive ECMO. Specifically, regional infrastructure disparities and varying physician perceptions across specialties create inequalities in care access. These results underscore the need for standardized rehabilitation protocols and targeted policy interventions to harmonize clinical practices and optimize the long-term functional recovery of ECMO survivors nationwide.

## Acknowledgments

We would like to express our gratitude to all those who contributed to this research. We sincerely appreciate the support and assistance of our colleagues throughout the process. Additionally, we acknowledge the valuable input from our advisors and reviewers. We would also like to extend our thanks to the institution for providing the necessary resources for this study.

## Author contributions

**Conceptualization:** Hyeng-Kyu Park, Hyun-Seok Jo, Min-Keun Song, Jae-Young Han, In-Sung Choi.

**Data curation:** Hyeng-Kyu Park, Hyun-Seok Jo, Min-Keun Song, Jae-Young Han, In-Sung Choi.

**Formal analysis:** Hyeng-Kyu Park, Hyun-Seok Jo, In-Sung Choi.

**Funding acquisition:** Hyun-Seok Jo, In-Sung Choi.

**Investigation:** Hyeng-Kyu Park, Hyun-Seok Jo, Min-Keun Song, Jae-Young Han.

**Methodology:** Hyeng-Kyu Park, Hyun-Seok Jo, Jae-Young Han.

**Project administration:** Hyun-Seok Jo.

**Resources:** Hyeng-Kyu Park, Jae-Young Han.

**Software:** Hyeng-Kyu Park.

**Supervision:** Hyeng-Kyu Park, Min-Keun Song, Jae-Young Han, In-Sung Choi.

**Validation:** Min-Keun Song.

**Visualization:** Hyeng-Kyu Park.

**Writing – original draft:** Hyeng-Kyu Park, Hyun-Seok Jo.

**Writing – review & editing:** Hyeng-Kyu Park.




