# Supplementary material for: Utilization and determinants of rehabilitation in patients receiving extracorporeal membrane oxygenation in South Korea (2014–2018): A study based on Health Insurance Review and Assessment (HIRA) data
Source: Medicine (Baltimore). 2026 Jun 5;105(23):e49196. doi: 10.1097/MD.0000000000049196 (PMC13246082; doi:10.1097/MD.0000000000049196)
Supplement: Supplementary file 1 [file medi-105-e49196-s001.docx]

**S1 Table. Claim codes for treatments or procedures**

| **Treatment/procedure** | **Health Insurance Review and Assessment claim codes** |
| --- | --- |
| Intensive care unit admission | AJ001-AJ590900 |
| Physical therapy | MM101, MM102, MM105, MM301, MM302 |
| Occupational therapy | MM111, MM112, MM113, MM114 |
| Mechanical ventilation | M5850, M5857, M5858, M5860 |
| Extracorporeal membrane oxygenation | O1903, O1904 |
| Hemodialysis | O7020, O7031-O7034, O7051-O7054, O7062 |
