# Supplementary material for: Utilization and determinants of rehabilitation in patients receiving extracorporeal membrane oxygenation in South Korea (2014–2018): A study based on Health Insurance Review and Assessment (HIRA) data
Source: Medicine (Baltimore). 2026 Jun 5;105(23):e49196. doi: 10.1097/MD.0000000000049196 (PMC13246082; doi:10.1097/MD.0000000000049196)
Supplement: Supplementary file 2 [file medi-105-e49196-s002.docx]

**S2 Table. Korean Drug and Anatomical Therapeutic Chemical Codes for treatments**

| **Drugs** | **Korean Drug and Anatomical Therapeutic Chemical Codes** |
| --- | --- |
| Vasopressors |  |
| Dopamine | 148701BIJ, 148702BIJ, 148703BIJ, 148704BIJ, 148731BIJ, 148732BIJ |
| Dobutamine | 148230BIJ, 148201BIJ |
| Norepinephrine | 203103BIJ, 203130BIJ, 203131BIJ, 203133BIJ, 203132BIJ, 203133BIJ |
| Sedatives |  |
| Midazolam | 195232BIJ, 195231BIJ, 195230BIJ, 195201BIJ, 195202BIJ, 195203BIJ, 195204BIJ |
| Fentanyl | 158301-158304/158331-15833/158311BIJ |
| Remifentanil | 457601BIJ, 457602BIJ, 457603BIJ |
| Ketamine | 179501BIJ, 179502 BIJ, 179503BIJ, 179530BIJ, 179531BIJ, 179532BIJ, 179533BIJ |
| Propofol | 219801-219808BIJ, 219830-219839BIJ |
| Morphine | 197201-197202/197230-197231/197301-197306/197330-197339/197340 BIJ |
| Neuromuscular blockers |  |
| Vecuronium | 247401-247402BIJ |
| Atracurium | 111601, 111630BIJ |
| Rocuronium | 224701-224702, 224730-224731 BIJ |
| Cis-atracurium | 359101-359104 BIJ, 359130-359131BIJ |
